# On the Antagonism of Atropia and Morphia, &c.

**Published:** 1865-09

**Authors:** Weir Mitchell


### Miscellaneous.

### On the Antagonism of Atropia and Morphia, &c.

BY DRS. WEIR MITCHELL, KEEN AND MOREHOUSE.

The foregoing experiments and observations authorize us, we think, to draw the following conclusions as to the use of hypodermic injections, and as to the antagonism of atropia and morphia:

1. Conia, atropia, and daturia have no power to lessen pain when used subdermally.

2. Morphia thus used is of the utmost value to relieve pain, and is most potent, in certain forms of neuralgia, the nearer it is applied to the seat of the suffering.

3. Morphia lowers the pulse slightly or not at all; atropia usually lowers the pulse a few beats within ten minutes, and then raises it twenty to fifty beats within an hour. The pulse finally falls about the tenth hour below the normal number, and regains its healthy rate within twenty-four hours.

4. Morphia has no power to prevent atropia from thus influencing the pulse, so that, as regards the circulation, they do not counteract one another.

5. During the change of the pulse under atropia, the number of respirations is hardly altered at all.

6. As regards the eye, the two agents in question are mutually antagonistic, but atropia continues to act for a much longer time than morphia.

7. The cerebral symptoms caused by either drug are, to a great extent, capable of being overcome by the other, but owing to the different rates at which they move to affect the system, it is not easy to obtain a perfect balance of effects, and this is made the more difficult from the fact already mentioned, that atropia has the greater duration of toxic activity.

8. The dry mouth of atropia is not made less by the coincident or precedent use of morphia. Atropia does not constipate, and may even relax the bowels; morphia has a reverse tendency.

9. The nausea of morphia is not antagonized or prevented by atropia.

10. Both agents cause dysuria in certain cases, nor is the dysuria occasioned by the one agent relieved by the other.

11. Atropia has no ability to alter or lessen the energy with which morphia acts to diminish sensibility or relieve the pain of neuralgic disease.

12. As regards toxic effects upon the cerebral organs, the two agents are mutually antidotal, but this antagonism does not prevail throughout the whole range of their influence, so that, in some respects, they do not counteract one another, while as concerns one organ, the bladder, both seem to affect it in a similar way.—*Amer. Jour. of Med. Sciences*, July, 1865.

